# Supplementary material for: Active site specificity profiling datasets of matrix metalloproteinases (MMPs) 1, 2, 3, 7, 8, 9, 12, 13 and 14
Source: Data Brief. 2016 Feb 22;7:299–310. doi: 10.1016/j.dib.2016.02.036 (PMC4777984; doi:10.1016/j.dib.2016.02.036)
Supplement: Supplementary file 10 — Supplementary material [file mmc10.zip › WebPICS_hMMP3_T_1%/P2prime.html]

 

PICS results


|  |  |
| --- | --- |
| **P2prime\_H**  6 in 199 sites   3.0 %    effects > 10 perc. pnts.  (vice-versa in brackets)  P2\_M: 13.2 (11.3)   P1prime\_M: 13.7 (13.7) |  |
| **P2prime\_I**  22 in 199 sites   11.1 %    effects > 10 perc. pnts.  (vice-versa in brackets)  P3\_C: 12.1 (88.9)   P2\_Q: 10.7 (15.6)   P1\_Q: 12.7 (25.3)   P1prime\_V: 16.2 (11.5) |  |
| **P2prime\_M**  7 in 199 sites   3.5 %    effects > 10 perc. pnts.  (vice-versa in brackets)  P2\_M: 39.4 (39.4)   P1\_F: 38.9 (34.0)   P1prime\_W: 11.3 (13.2) |  |
| **P2prime\_Q**  11 in 199 sites   5.5 %    effects > 10 perc. pnts.  (vice-versa in brackets)  P3\_H: 15.2 (27.8)   P1prime\_Q: 30.9 (30.9)   P3prime\_H: 15.2 (27.8)   P3prime\_Q: 12.2 (11.2) |  |
| **P2prime\_T**  20 in 199 sites   10.1 %    effects > 10 perc. pnts.  (vice-versa in brackets)  P3\_L: 13.4 (11.6) |  |
| **P2prime\_V**  26 in 199 sites   13.1 %    effects > 10 perc. pnts.  (vice-versa in brackets)  P1\_N: 14.8 (16.1) |  |
